# Human lung tissue provides highly relevant data about efficacy of new anti-asthmatic drugs

**DOI:** 10.1371/journal.pone.0207767

**Published:** 2018-11-30

**Authors:** Olga Danov, Sharon Melissa Jiménez Delgado, Helena Obernolte, Sophie Seehase, Susann Dehmel, Peter Braubach, Hans-Gerd Fieguth, Gabriele Matschiner, Mary Fitzgerald, Danny Jonigk, Sascha Knauf, Olaf Pfennig, Gregor Warnecke, Judy Wichmann, Armin Braun, Katherina Sewald

**Affiliations:** 1 Fraunhofer Institute for Toxicology and Experimental Medicine ITEM, Biomedical Research in Endstage and Obstructive Lung Disease Hannover (BREATH), Member of the German Center for Lung Research (DZL), Hannover, Germany; 2 Research Center Borstel, Airway Research Center North (ARCN), Member of the German Center for Lung Research (DZL), Borstel, Germany; 3 Institute for Pathology, Hannover Medical School, Biomedical Research in Endstage and Obstructive Lung Disease Hannover (BREATH), Member of the German Center for Lung Research (DZL), Hannover, Germany; 4 KRH Klinikum Siloah-Oststadt-Heidehaus, Hannover, Germany; 5 Pieris AG, Freising-Weihenstephan, Germany; 6 Pathology Unit, German Primate Center, Leibniz-Institute, Göttingen, Germany; 7 Division of Cardiac, Thoracic, Transplantation, and Vascular Surgery, Hannover Medical School, Biomedical Research in Endstage and Obstructive Lung Disease Hannover (BREATH), Member of the German Center for Lung Research (DZL), Hannover, Germany; 8 Institute of Immunology, Hannover Medical School, Hannover, Germany; National Jewish Health, UNITED STATES

## Abstract

Subgroups of patients with severe asthma are insensitive to inhaled corticosteroids and require novel therapies on top of standard medical care. IL-13 is considered one of the key cytokines in the asthma pathogenesis, however, the effect of IL-13 was mostly studied in rodents. This study aimed to assess IL-13 effect in human lung tissue for the development of targeted therapy approaches such as inhibition of soluble IL-13 or its receptor IL-4Rα subunit. Precision-cut lung slices (PCLS) were prepared from lungs of rodents, non-human primates (NHP) and humans. Direct effect of IL-13 on human lung tissue was observed on inflammation, induction of mucin5AC, and airway constriction induced by methacholine and visualized by videomicroscopy. Anti-inflammatory treatment was evaluated by co-incubation of IL-13 with increasing concentrations of IL-13/IL-13 receptor inhibitors. IL-13 induced a two-fold increase in mucin5AC secretion in human bronchial tissue. Additionally, IL-13 induced release of proinflammatory cytokines eotaxin-3 and TARC in human PCLS. Anti-inflammatory treatment with four different inhibitors acting either on the IL-13 ligand itself (anti-IL-13 antibody, similar to Lebrikizumab) or the IL-4Rα chain of the IL-13/IL-4 receptor complex (anti-IL-4Rα #1, similar to AMG 317, and #2, similar to REGN668) and #3 PRS-060 (a novel anticalin directed against this receptor) could significantly attenuate IL-13 induced inflammation. Contrary to this, IL-13 did not induce airway hyperresponsiveness (AHR) in human and NHP PCLS, although it was effective in rodent PCLS. Overall, this study demonstrates that IL-13 stimulation induces production of mucus and biomarkers of allergic inflammation in human lung tissue *ex-vivo* but no airway hyperresponsiveness. The results of this study show a more distinct efficacy than known from animals models and a clear discrepancy in AHR induction. Moreover, it allows a translational approach in inhibitor profiling in human lung tissue.

## Introduction

In industrialized countries, the incidence of allergic asthma has rapidly increased in the second half of the 20^th^ century, affecting approximately 4 to 5% of the population worldwide. Allergic asthma is mediated by activated T_H_-2 cells promoting lung inflammation by secretion of key cytokines such as IL-4, IL-5, and IL-13 [1, 2]. These cytokines are elevated in bronchoalveolar lavage (BAL) fluid of asthmatic patients after allergen challenge [3–5]. IL-4 and IL-13 in particular are closely related pleiotropic cytokines with overlapping functions [6]. However, IL-13 has been shown to be a more relevant mediator of allergic asthma [7, 8]. IL-13 is secreted not only by activated T_H_-2 cells, but also by mast cells, basophils, eosinophils, and type-2 innate lymphoid cells (ILC2s). Its main function includes mediation of lymphocytic and eosinophilic inflammation, goblet cell hyperplasia, and mucus production [7–10]. Moreover, it is crucial for differentiation of B-cells into IgE-producing plasma cells, which ultimately results in a switch of the immunoglobulin isotype class. It has also been hypothesized that IL-13 strongly contributed to bronchial hyperresponsiveness [11].

The heterodimeric transmembrane receptor is formed by IL-13 that binds IL-13 receptor α1 chain (IL-13Rα1) and recruits IL-4 receptor α chain (IL-4Rα) to form the IL-4/IL-13 type-II receptor complex [12]. This receptor complex is shared by both cytokines, IL-4 and IL-13.

Receptor activation by binding of IL-4 or IL-13 initiates selective upregulation of the CC-chemokines eotaxin-2/3 (CCL24/CCL26) and thymus and activation-regulated chemokine (TARC/CCL17). The role of these chemokines is to recruit inflammatory cells such as eosinophils via chemokine receptor CCR3. Eotaxin-3 and TARC are potential biomarkers for anti-IL-13 therapeutics [13]. Cytokines and the subsequently induced airway inflammation contribute to airway hyperresponsivess.

Substantial effort has been made to develop successful therapies to treat asthma. Nowadays, inhaled glucocorticoids, often in combination with long-acting β2 agonists, are widely used therapeutics. Yet, side-effects and reduced effectiveness in steroid-resistent patients [14, 15] highlight the unmet medical need for the development of novel therapies. In the past years, there has been extensive research on the inhibition of disease-related pathways. As IL-13 was shown to be critical in asthma pathogenesis, it is an attractive target for pharmacological intervention. The reduction or blocking of IL-13-mediated pathological effects can be beneficial for asthma patients. So far, several monoclonal antibodies have been developed to neutralize IL-13 effects in asthmatic patients. Anti-IL-13 monoclonal antibody (Lebrikizumab) from Genentech (Member of the Roche Group, Basel, Switzerland) leads to neutralization of IL-13-related pharmacodynamic biomarkers, although its effect on significant reduction in asthma exacerbations was not consistent, especially in biomarker-high patients [16, 17]. Other approaches use monoclonal antibodies targeting soluble IL-13 or the IL-4Rα chain, leading to simultaneous inhibition of both IL-4 and IL-13 pathways. Corren and colleagues have tested the monoclonal IgG_2_ antibody AMG 317 from Amgen (Thousand Oaks, CA, USA) binding to IL-4Rα in a phase-II clinical trial in patients with moderate to severe asthma. AMG 317 did not demonstrate statistically significant change in Asthma Control Questionnaire symptom score which is proposed as the primary endpoint in asthma clinical trials, although AMG 317 showed decreased numbers of exacerbations [18]. In contrast, another IL-4Rα monoclonal antibody (mAb) REGN668 (Dupilumab/SAR231893) developed by Regeneron (Tarrytown, USA) in collaboration with Sanofi (Paris, France) revealed clinical efficacy in reducing asthma exacerbations, improving lung function and symptom control in patients with persistent moderate to severe eosinophilic asthma and is currently in a phase-III clinical trial (NCT02414854) [19].

An interesting and novel alternative to monoclonal antibodies are therapeutic biological molecules that can target cytokine receptors such as Anticalin proteins or Fab fragments. Anticalin proteins are artificial lipocalins folded as rigid β-barrels with eight antiparallel β-strands forming four hypervariable loops as a scaffold for the antigen binding site. The advantages of using engineered Anticalin proteins instead of mAb are their potency, selectivity, small size, the cost-saving expression system (microbial instead of mammalian cells) and the fact that they are built as a single polypeptide chain providing extra robustness. Moreover, mutation of the binding site can improve the potency and selectivity of the antagonists in the discovery phase [20–22]. Additionally, Anticalin proteins can be delivered via the inhaled route at much lower predicted doses than systemically applied antibodies.

Most of the mechanistic understanding of IL-13 was gained in *in-vitro* and *in-vivo* animal models of allergic asthma. Here, we provide data concerning the activity of IL-13 on human lung tissue *ex-vivo*. The aim of this study was to characterize the effect of IL-13 on human lung tissue *ex-vivo* based on defined clinically relevant biomarkers and to test the efficacy of newly developed inhibitors against IL-13-induced effector mechanisms. In this study, precious human data are provided that supplement preclinical data from *in-vitro* and *in-vivo* animal studies and may help to estimate the efficacy of new drugs in humans.

## Materials and methods

### Ethics statement

Human lung lobes were acquired from patients who underwent lobe resection for cancer at Hannover Medical School (MHH, Hannover, Germany) or KRH Klinikum Siloah-Oststadt-Heidehaus (Hannover, Germany). These experiments were approved by the ethics committee of the Hannover Medical School (MHH, Hannover, Germany) and are in compliance with *The Code of Ethics of the World Medical Association* (renewed on 2015/04/22, number 2701–2015). All patients or their next of kin gave written informed consent for the use of their lung tissue for research.

Common marmoset (*Callithrix jacchus*) lung tissue originated from respiratory-healthy animals and was obtained from the German Primate Center (Göttingen, Germany). Housing conditions were in line with German and European regulations: the German Animal Protection Act (Tierschutzgesetz, Tierschutz-Versuchstierverordnung) and the European Directive on the protection of animals used for scientific purposes (2010/63/EU). The Animal Welfare Officer and Ethics Committee of the German Primate Center approved the use of samples for this study.

### Media, chemicals and reagents

Dulbecco’s modified Eagle’s medium/nutrient mixture F-12 Ham (DMEM, pH 7.2–7.4) with L-glutamine and 15 mM HEPES without phenol red and fetal bovine serum was purchased from Gibco (Life technologies, Darmstadt, Germany). Medium for cultivation was supplemented with 100 units/mL penicillin and streptomycin, which were purchased from Lonza (Verviers, Belgium). Medium for airway constriction measurements was additionally supplemented with 25 mM HEPES, supplied by Lonza (Verviers, Belgium). Dulbecco´s phosphate-buffered solution without Ca^2+^ and Mg^2+^ (DPBS) was also purchased from Lonza (Verviers, Belgium). Low gelling temperature agarose, Earle’s Balanced Salt Solution (EBSS), protease inhibitor cocktail P1860, and Triton X-100 were supplied by Sigma-Aldrich (Munich, Germany). WST-1 assay kit was obtained from Roche (Mannheim, Germany) and Pierce BCA Protein Assay Kit was supplied by Thermo Scientific (Rockford, IL, USA). Mouse, rat, and human IL-13 and human IL-13 Variant were obtained from PeproTech (Hamburg, Germany). Antibodies used for immunofluoresence staining were rabbit IL-13Rα1 (ab79277) and rabbit mucin5AC (ab78660) obtained from Abcam (Cambridge, UK), mouse pan-cytokeratin (C2562), rabbit IgG_1_ (I5006) obtained from Sigma-Aldrich (Munich, Germany), mouse IgG_1_ and mouse IgG_2a_ (X0931/X0943) purchased from Dako (Glostrup, Denmark) as well as donkey-anti-rabbit Cy3 (711-166-152), donkey-anti mouse Alexa 647 (715-606-151) and donkey serum supplied by Dianova GmbH (Hamburg, Germany). To-Pro-3 was purchased from Invitrogen (Life technologies, Darmstadt, Germany). The original inhibitors for evaluation in human PCLS used in this study were commercially not available. Therefore, the inhibitors were redesigned and synthesized by Pieris Pharmaceuticals based on the patent bulletine sequences: monoclonal anti-IL-13 Ab (similar to Lebrikizumab), anti-IL-4Rα #1 (similar to AMG 317), anti-IL-4Rα #2 (similar to REGN668), and anti-IL-4Rα #3 (Anticalin PRS-060). Tlc26, the backbone structure upon which PRS-060 is based, was used as a negative control for anti-IL-4Rα #3.

### Animals and husbandry conditions

Female mice (Balb/c, 10–12 weeks) and rats (Brown Norway, 10–12 weeks) were obtained from Charles River (Sulzfeld, Germany). Animals were kept under conventional housing conditions (22°C, 55% humidity, and 12-h day/night rhythm). Mice and rats were sacrificed by an overdose of pentobarbital sodium (Narkoren, Merial GmbH, Hallbergmoos, Germany) injected *i*.*p*.

Common marmosets (3–9 years old) were kept pairwise under conventional housing conditions (25°C, 60–80% humidity, and 12-h day/night cycle). Marmosets were anesthetized by using diazepam (Ratiopharm, Ulm, Germany) and alfaxalone (Alphaxan, Jurox (UK) Limited, Worcestershire, United Kindom), followed by an intracardial lethal dose of pentobarbital sodium (Narkoren, Merial GmbH, Hallbergmoos, Germany) under deep general anaesthesia.

### Human lung tissue

Lung tissue acquired from patients who were diverse in gender, age, regarding their disease background, medications, and life style. Patients were anonymised. Their allergy status, medication, other diseases, and life style were unknown. Only tissue from macroscopically and microscopically healthy parts of the lung were used for experiments. Each experiment was conducted with positive and negative controls for each individual assay providing information about the viability and responsiveness of the tissue.

### Preparation of PCLS

Mouse, rat and marmoset [23–25] as well as human [26, 27] lung slices were prepared as described previously. Briefly, mouse lungs were filled *post mortem in situ*, whereas rat, marmoset and human lungs were filled *ex situ*. Lungs were inflated with 37°C warm 1.5% (wt/vol) low melting agarose/DMEM solution and cooled on ice until agarose polymerization.

Mouse lungs were sliced into approximately 300 μm thin sections in 4°C cold EBSS using an Automatic Oscillating Tissue Slicer (OTS 5000, Warner Instruments). Rat, marmoset and human PCLS were prepared from cylindrical tissue cores of 8 mm diameter with a centered airway (for airway constriction measurements) and cut into approximately 300 μm thick slices using a Krumdieck tissue slicer (Alabama Research and Development, Muniford, AL, USA) in EBSS. Tissue slices were transferred into petri dishes and washed with DMEM for 2–3 h in order to remove cell debris.

### Stimulation of PCLS for cytokine release

Two PCLS per well were treated in duplicates with 1–100 nM (1 nM; 10 nM; 25 nM; 50 nM; 100 nM) of IL-13 in 500 μL DMEM for 24 h under standard cell culture conditions (37°C, 5% CO_2_, 100% humidity). To inhibit cytokine release, 10 nM IL-13 were co-incubated with different concentrations of IL-13 inhibitors (1 nM; 3 nM; 10 nM; 30 nM; 100 nM). To this end, anti-IL-13 Ab, anti-IL-4Rα #1 Ab, anti-IL-4Rα #2 Ab, and anti-IL-4Rα #3 were used to inhibit IL-13-induced effects. Untreated PCLS were used in all experiments as negative controls.

After 24-h incubation, supernatants were collected and supplemented with 0.2% protease inhibitor cocktail. Afterwards, PCLS were permeabilized with 1% Triton X-100 in DPBS supplemented with 0.2% protease inhibitor cocktail for 1 h at 4°C. Supernatant and lysis extract were stored at -80°C for further analysis.

### Protein determination

The protein amount in PCLS was determined using Pierce BCA Protein Assay Kit according to the manufacturer’s instructions. Twenty-five μL of lysate were mixed with 200 μL of reaction mixture, incubated for 30 min at 37°C and measured in duplicates at 570 nm in a microplate reader.

### Measurement of T_H_-2 induced cytokines (ELISA)

Eotaxin-3 and TARC were measured in duplicates by commercially available ELISA according to the manufacturer’s specifications (ELISA Duoset, purchased from R&D Systems, Wiesbaden, Germany). For detection, 3,3’,5,5’-tetramethylbenzidine (TMB, Kem-En-Tec diagnostics, Cologne, Germany) was used. Samples were measured at 450 nm with a reference wavelength of 570 nm. For comparison of cytokine release in different samples and patients the total amount of released cytokine (pg) was normalized to mg of protein content in PCLS.

### Airway constriction measurement in PCLS

Airway constriction was measured as described previously [28]. Briefly, PCLS with peripheral airways (500 μm to 1300 μm) selected for airway constriction measurement had intact full smooth-muscle layers, visible regular cilia beating, comparable airway size, sustained tissue viability, and responsiveness to controls. PCLS were incubated with 8 nM (equal to 100 ng/mL) of IL-13 of corresponding species (for marmoset rhIL-13 was used) for 0.5, 3, 6, and 18 h, followed by methacholine challenge with different doses from 10^−10^ to 10^−3^ M to obtain a cumulative dose-response curve. From every lung, we performed repeated measurements (two technical replicates per time point and minimum three biological replicates) with constant temperature and duration. Single tissue sections were placed in a 6-well cell culture plate and fixed by a nylon thread to prevent shifting during the measurement. Tissue was kept at 37°C and imaged by microscope and a CCD camera AxioCam ICm1 (Stereo microscope Zeiss Discovery V8, Zeiss, Jena, Germany) every five seconds for a period of five minutes until the next higher concentration of methacholine was added.

Analysis of airway constriction measurement was performed with ImageJ (ImageJ 1.49, national Institute of Health, USA) using the area selection tool. The airway area prior to methacholine administration was defined as reference corresponding to 100% (baseline). The minimum of the airway area after addition of each methacholine concentration was calculated as a percentage of the reference. EC_50_ in response to methacholine administration was calculated by sigmoidal dose-response curve fitting using the GraphPad Prism 4.03 software (GraphPad software, San Diego, CA, USA).

### Mucus overproduction in human bronchus

A human bronchus was cut manually into equal tissue sections of about 0.1 cm^2^. Tissue sections were then weighed and subsequently washed 4 times for 2 h after preparation. Two bronchus pieces per well were cultured in medium or medium containing 10 nM of IL-13 for 20 h at 37°C. After the incubation period the culture supernatant was supplemented with 0.2% protease inhibitor cocktail and stored at -80°C for detection by ELISA. The mucin5AC ELISA was prepared in duplicates according to the manufacturer’s specifications (Cusabio, CSB-E10109h). Samples were measured at 450 nm with a reference wavelength at 570 nm.

### Pas staining of human airways

Formalin-fixed non-asthmatic human lung tissue was embedded in paraffin and cut in 4 μm thin sections. After deparaffinization and rehydration, slices were stained with periodic acid-Schiff (PAS) to reveal mucus production according to standard procedures. As previously described [29], PAS-positive cells were counted manually. The number of cells was normalized to the lenght on the basal side of the bronchial epithelial perimeter (PAS-positive cells per mm basement membrane).

### Immunofluorescence staining of the IL-13 receptor in PCLS

PCLS were fixed in 2% paraformaldehyde in DPBS overnight at 4°C. After permeabilization with 0.3% Triton X-100 in DPBS and saturation with 4% donkey serum in DPBS for 30 min at room temperature, slices were incubated overnight with primary antibody rabbit IL-13Rα1 (diluted 1:100) and rabbit IgG_1_ (diluted 1:1,000) for isotype control. Afterwards, PCLS were washed with 0.05% Tween in DPBS for 6 h, with medium replacement on an hourly basis, and blocked again with 4% donkey serum in DPBS for 30 min at room temperature. Tissue sections were incubated with secondary antibody donkey-anti-rabbit Cy3 (diluted 1:800) overnight at 4°C. Specific staining of cell nuclei was performed with 1.3 μg/mL To-Pro-3 diluted in 4% donkey serum for 10 min at room temperature. Immunofluorescence imaging was performed using a confocal laser scanning microscope Meta 510 (Zeiss, Jena, Germany) equipped with an argon laser and a helium/neon-laser illumination system. PCLS were measured with the 40x water immersion objective and an excitation wavelength of 543 nm using an emission filter LP 560–600 nm. The staining of IL-13 receptor α1 was compared with the IgG_1_ isotype control.

### Immunofluorescence staining of mucin5AC in bronchial tissue

Bronchial sections were fixed and stained as described above for PCLS. Mucin5AC was stained with primary polyclonal antibody rabbit mucin5AC (diluted 1:200) and rabbit IgG (diluted 1:1,000) for isotype control. For epithelial cell staining monoclonal mouse pan-cytokeratin (diluted 1:100) and corresponding isotype controls mouse IgG_1_ and mouse IgG_2a_ (diluted 1:100) were used. Donkey-anti-rabbit Cy3 (diluted 1:800) and donkey-anti mouse Alexa 647 (diluted 1:800) were used as secondary antibodies.

### Statistical analysis

The number of human individual donors or animals (n) is indicated in each figure legend (biological replicates). We reported all data that were available at the time of publication. We did not remove data and did not reduce the number of donors to equalize data sets.

Statistical analysis was performed using GraphPad Prism 4.03 (GraphPad Software, Inc., La Jolla, USA). Mucus hypersecretion was assessed using Wilcoxon signed-rank test. ELISA data were analyzed using Friedman test and Dunn’s Multiple Comparison test. Airway constriction analysis was performed by applying a one-way ANOVA with Dunnett's Multiple Comparison test. All values in figures are given as mean ± SEM and considered statistically significant, if p ≤ 0.05.

## Results

### Fluorescent staining of IL-13 receptor α1 in PCLS

**Fig 1:**
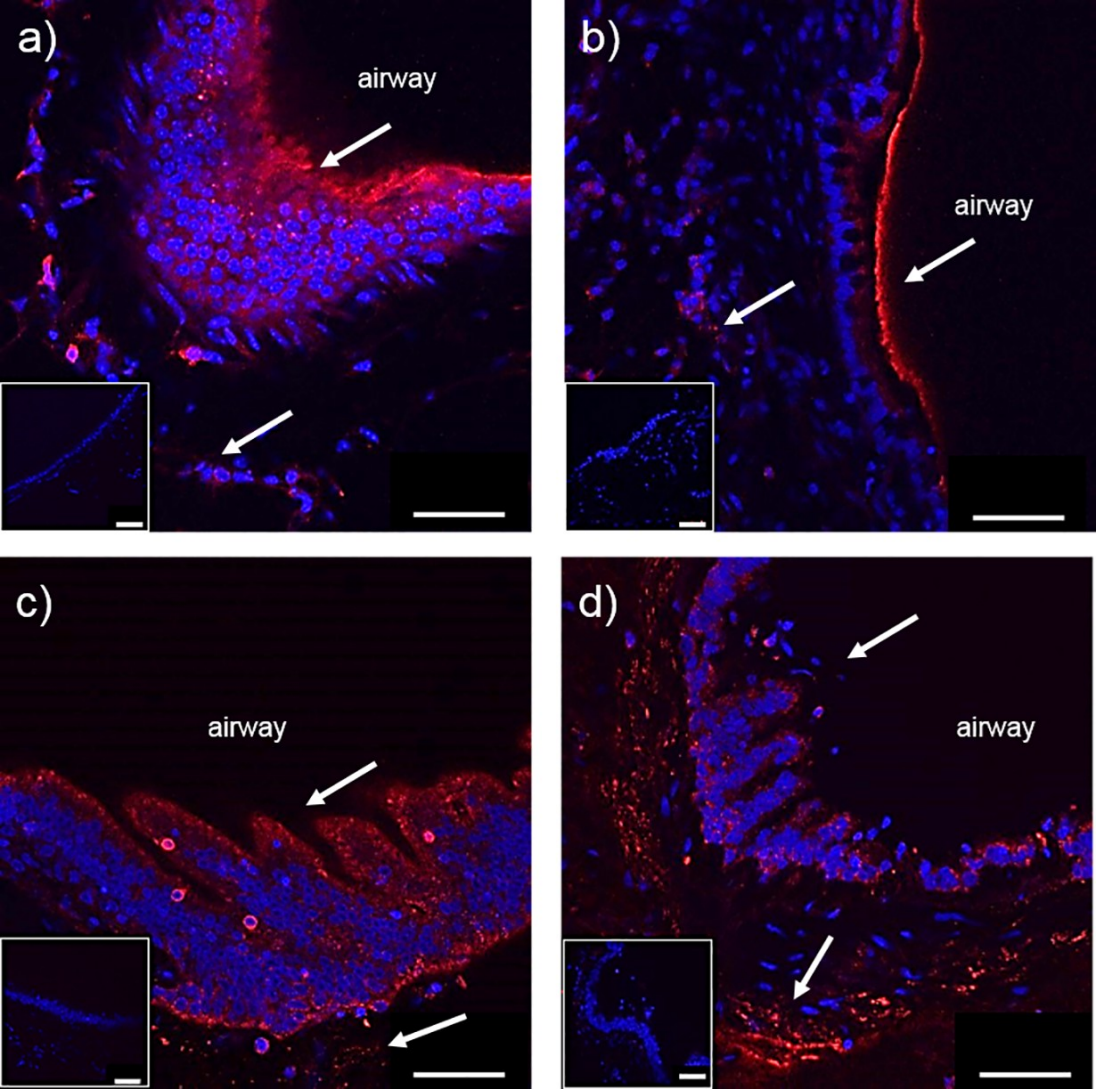
**Representative fluorescence immunostaining of IL-13Rα1 in a) mouse, b) rat, c) marmoset, and d) human PCLS.** Red color shows IL-13 receptor α1 labeled with Cy3 and blue color shows cell nuclei stained with To-Pro-3. White arrows show the receptor presence in the airway epithelium and smooth muscle cells in human PCLS. The corresponding IgG isotype controls are shown in the inset photos. Scale bar: 50 μm.

IL-13 receptor α1 (IL-13Rα1) has been reported to be expressed on different rodent as well as human cells. In order to prove receptor expression and localization within the PCLS and to confirm a specific IL-13 triggered response, immunostaining of the receptor subunit was performed in all tested species. The confocal images in Fig 1 show specific staining of IL-13 receptor α1 in PCLS. For the morphological orientation of the cells in PCLS, epithelial and smooth muscle cells were indicated by an arrow and this confirms a receptor localization in all tested species (Fig 1).

### IL-13 induced mucin production in bronchial tissue sections

Hypersecretion of mucus is one of the three main pathophysiological changes in asthmatic patients, where mainly goblet cells and submucosal gland cells contribute to hypersecretion of mucins into the airways. The distribution of goblet cells was assessed in large to small airways. We found that the frequency of goblet cells was constant in different airway generations (S1C Fig) whereas the total number of goblet cells decreases with the airway size as was shown before [30].

Due to the lower total number of goblet cells in smaller airways, mucin5AC was only detectable in bronchial tissue but not in PCLS containing airways. Stimulation of human bronchial tissue with IL-13 induced a two-fold increase in mucin5AC protein after 20 h (S1A Fig). Location of mucin5AC was verified by confocal microscopy (S1B Fig). This data shows that IL-13 is directly leading to mucus hypersecretion in bronchial tissue.

### IL-13 induced release of cytokines but not airway hyperresponsiveness in lung sections

**Fig 2:**
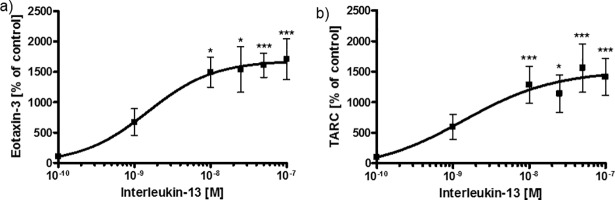
IL-13-induced eotaxin-3 and TARC secretion in human PCLS. Lung tissue was stimulated with human IL-13 for 24 h. a) Eotaxin-3 and b) TARC were measured in supernatant and tissue lysate and normalized (as a percentage) to the untreated tissue control. Data are presented as mean ± SEM; group sizes: a) n = 7 and b) n = 8; *p < 0.05, **p < 0.01; ***p < 0.001 compared with untreated tissue control (Friedman test and Dunn’s Multiple Comparison Post-hoc test).

In order to evaluate asthma-related biomarkers *ex-vivo*, the effects of IL-13 on eotaxin-3 and TARC secretion were measured in human PCLS in a dose-dependent manner. Optimal time points for cytokine release and AHR were assessed in kinetic experiments. Eotaxin-1 was not detectable in PCLS and eotaxin-2 showed comparable results to eotaxin-3 upon IL-13 stimulation (data not shown). Both TARC and eotaxin-3 were significantly elevated 24 h after stimulation with IL-13 in doses from 10 nM to 100 nM (Fig 2) and were not further increased after 48 h (data not shown). Stimulation with 10 nM of IL-13 resulted in a 15-fold increase of eotaxin-3 and a 13-fold increase of TARC secretion. Longer incubation periods did not lead to significantly higher cytokine levels, but higher concentrations of IL-13 induced eotaxin-3 and TARC secretion at top levels of the saturation curves. Absolute values of IL-13-induced eotaxin-3 (260±117 pg/mg vs. 4366±4111 pg/mg) and TARC (83±41 pg/mg vs. 965±811 pg/mg) secretion at 10 nM IL-13 are shown in S2 Fig. Therefore, 10 nM of IL-13 was selected for the profiling of IL-13 inhibitors.

**Fig 3:**
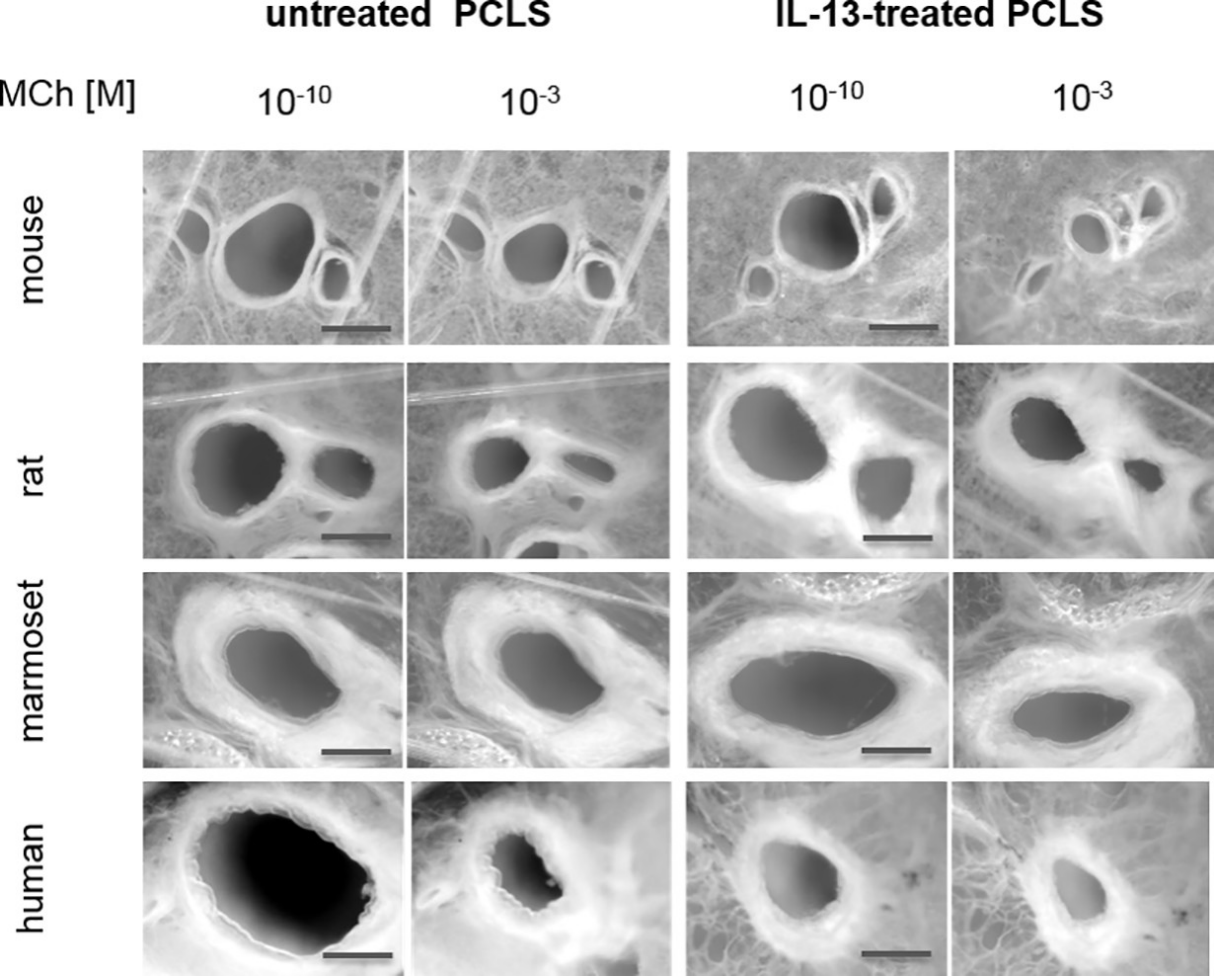
Methacholine-induced airway constriction in mouse, rat, marmoset, and human PCLS compared with pre-incubation with IL-13 of the corresponding species (except for marmoset, rhIL-13 was used). Macroscopic images of precision-cut lung slices of untreated PCLS (control) or PCLS treated with 8 nM of IL-13 for six hours prior to stimulation with 10^−10^ M and 10^−3^ M methacholine (MCh), the lowest and the highest concentration, respectively. Scale bar: 500 μm.

**Fig 4:**
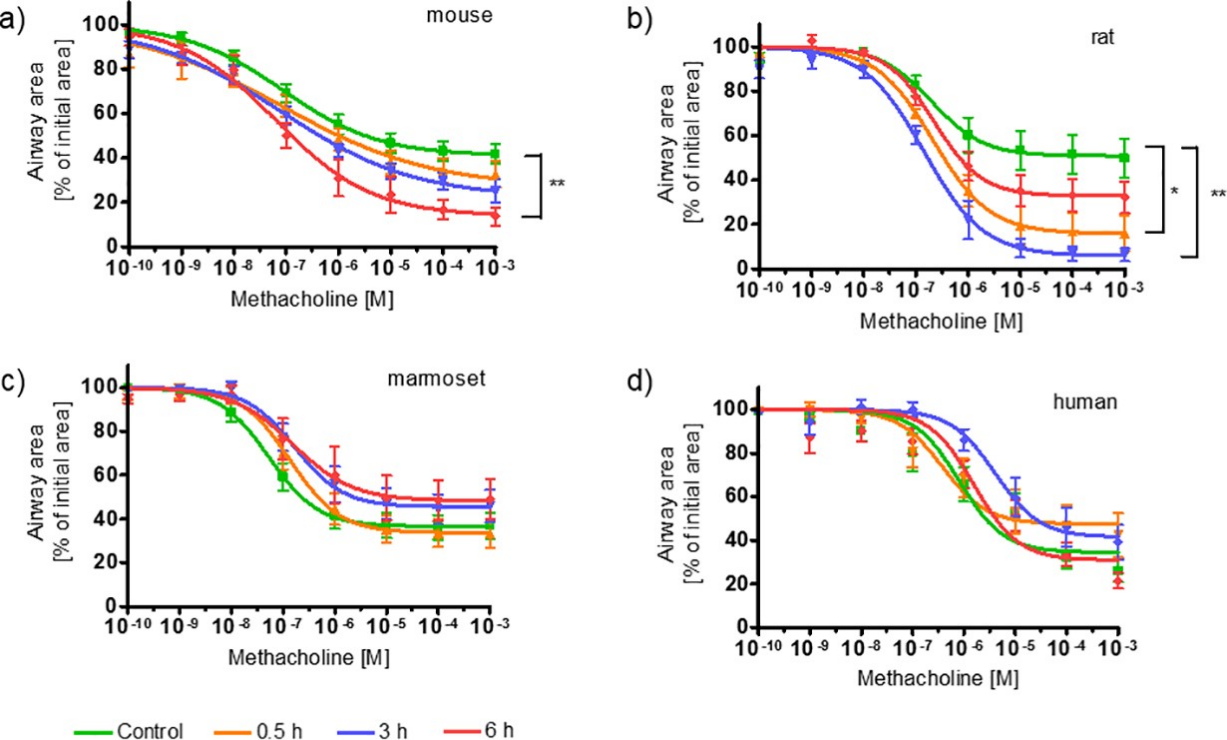
**Effect of IL-13 on methacholine-induced airway constriction in a) murine, b) rat, c) marmoset, and d) human PCLS.** PCLS were pre-incubated with 8 nM IL-13 for 0.5 h, 3 h, and 6 h before stimulation with increasing concentrations of methacholine. Airway constriction was determined as a percentage of the initial airway area. Data are presented as mean ± SEM; group sizes: a) n = 3; b) n = 3; c) n = 5 and d) n = 7; *p≤0.05; **p≤0.01 (one-way ANOVA Dunnett’s multiple comparison test vs. control group).

As the present study showed a significant increase in IL-13-mediated mucin5AC, eotaxin-3 and TARC secretion, it was further hypothezised that there is also a direct or indirect effect on the sensitivity of smooth muscle cells around airways. Therefore, IL-13-induced effects on airway smooth muscle cells leading to AHR were tested in human lung slices. A previously published study by Cooper *et al*. showed IL-13-induced AHR in human PCLS [31]. To verify this, concentration-dependent methacholine (MCh) challenge was performed to induce airway constriction in human PCLS. In our hands IL-13 treatment did not induce AHR to methacholine at those time points (18 h) in human PCLS (S3D Fig). The AHR showed no kinetic dependency until 24 h. Time points after 24 h were not assessed. Given that rodents and non-human pimates are used extensively in asthma research, IL-13 effects on AHR were investigated under the same conditions. MCh induced concentration-dependent airway constriction in all tested species. Pre-incubation with IL-13 for 18 h, however, did not enhance this effect (S3A–S3C Fig). We performed kinetic experiments with shorter incubation periods. Examples of macroscopic airway images of untreated PCLS and of PCLS treated with IL-13 for 6 h are shown in Fig 3. Shorter pre-incubations of 0.5 h, 3 h, and 6 h of mouse and rat PCLS with IL-13 resulted in similar EC_50_ values but in an increased maximum of airway constriction (C_max_) in response to MCh challenge (Fig 4). Hereby, C_max_ increased by 25% in murine PCLS after IL-13 pre-incubation of 6 h, and 45% greater airway constriction after 3 h of IL-13 pre-incubation compared with the control in rat PCLS (Table 1). Marmoset PCLS, similar to human PCLS, did not show AHR at any time point.

**Table 1:** Methacholine-induced airway constriction in the control group and in PCLS pre-treated with IL-13 (8 nM) for 0.5 h, 3 h and 6 h.

		Time of incubation with 8 nM IL-13		
	Control	0.5 h	3 h	6 h
**Mouse**				
EC_50_ [nM] C_max_ [%]	87 59 ± 3	83 73 ± 8	79 78 ± 4	64 86 ± 4**
**Rat**				
EC_50_ [nM] C_max_ [%]	197 49 ± 4	212 84 ± 4	142 95 ± 3**	220 69 ± 4
**Marmoset**				
EC_50_ [nM] C_max_ [%]	53 63 ± 3	137 66 ± 3	167 55 ± 3	153 53 ± 6
**Human**				
EC_50_ [nM] C_max_ [%]	812 65 ± 4	380 52 ± 4	4055 58 ± 5	1567 69 ± 4

Half-maximal effective concentrations (EC_50_) and maximal airway constriction (C_max_) were calculated for every species and every time point.

**p≤0.01 (one-way ANOVA Dunnett’s multiple comparison test vs. control group).

### Inhibition of eotaxin-3 and TARC release

**Fig 5:**
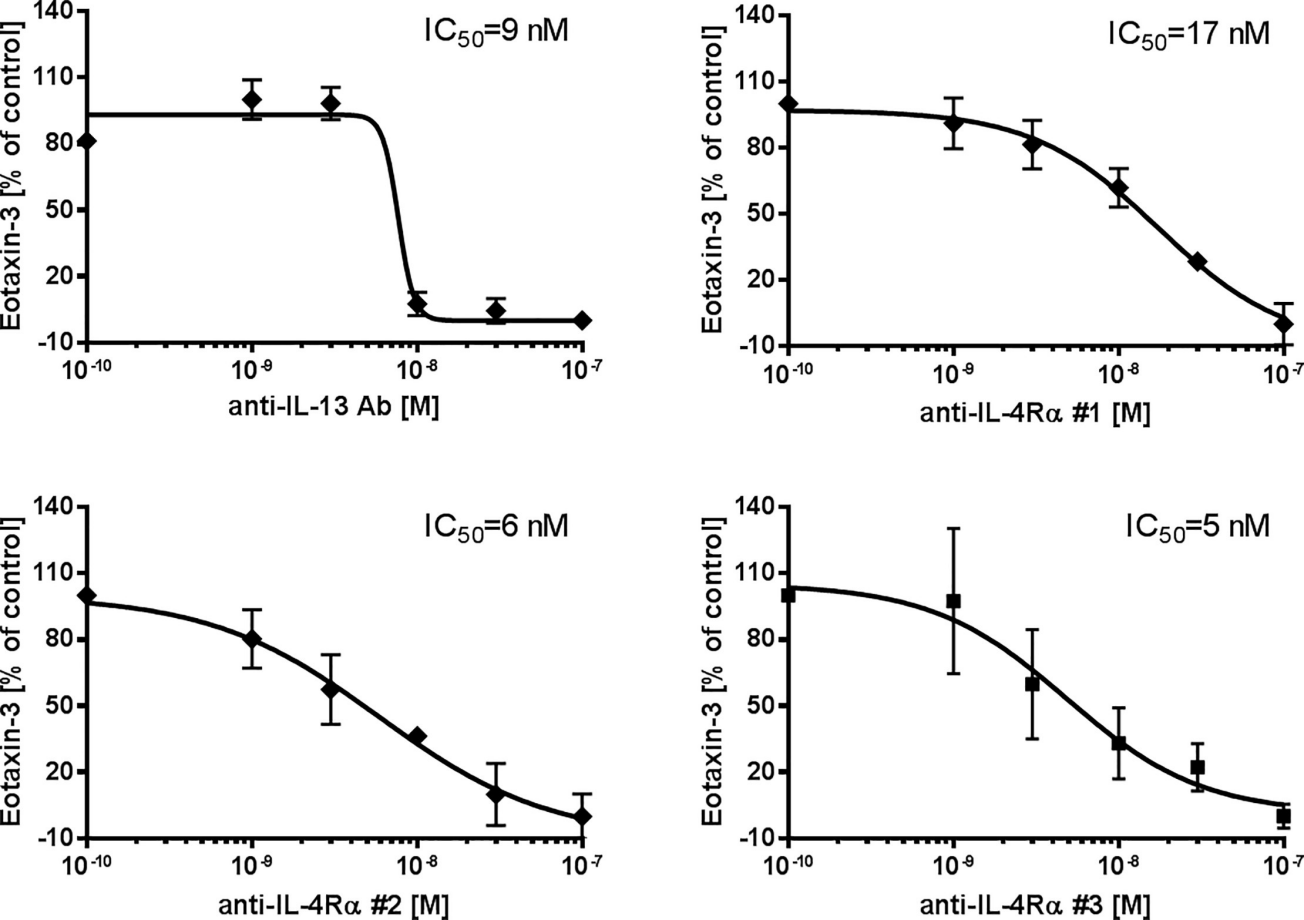
Inhibitory effects of four antagonists on IL-13-induced eotaxin-3 secretion. Human PCLS were co-stimulated with 10 nM of IL-13 and increasing concentrations of the antagonists a) anti-IL-13 Ab (n = 6), b) anti-IL-4Rα #1 (n = 5), c) anti-IL-4Rα #2 (n = 5), and d) anti-IL-4Rα #3 (n = 4). Total eotaxin-3 secretion was determined in supernatant and tissue lysate and normalized (as a percentage) to the tissue control. Curve fitting was performed by non-linear regression analysis based on four parameters. Data are presented as mean ± SEM.

**Fig 6:**
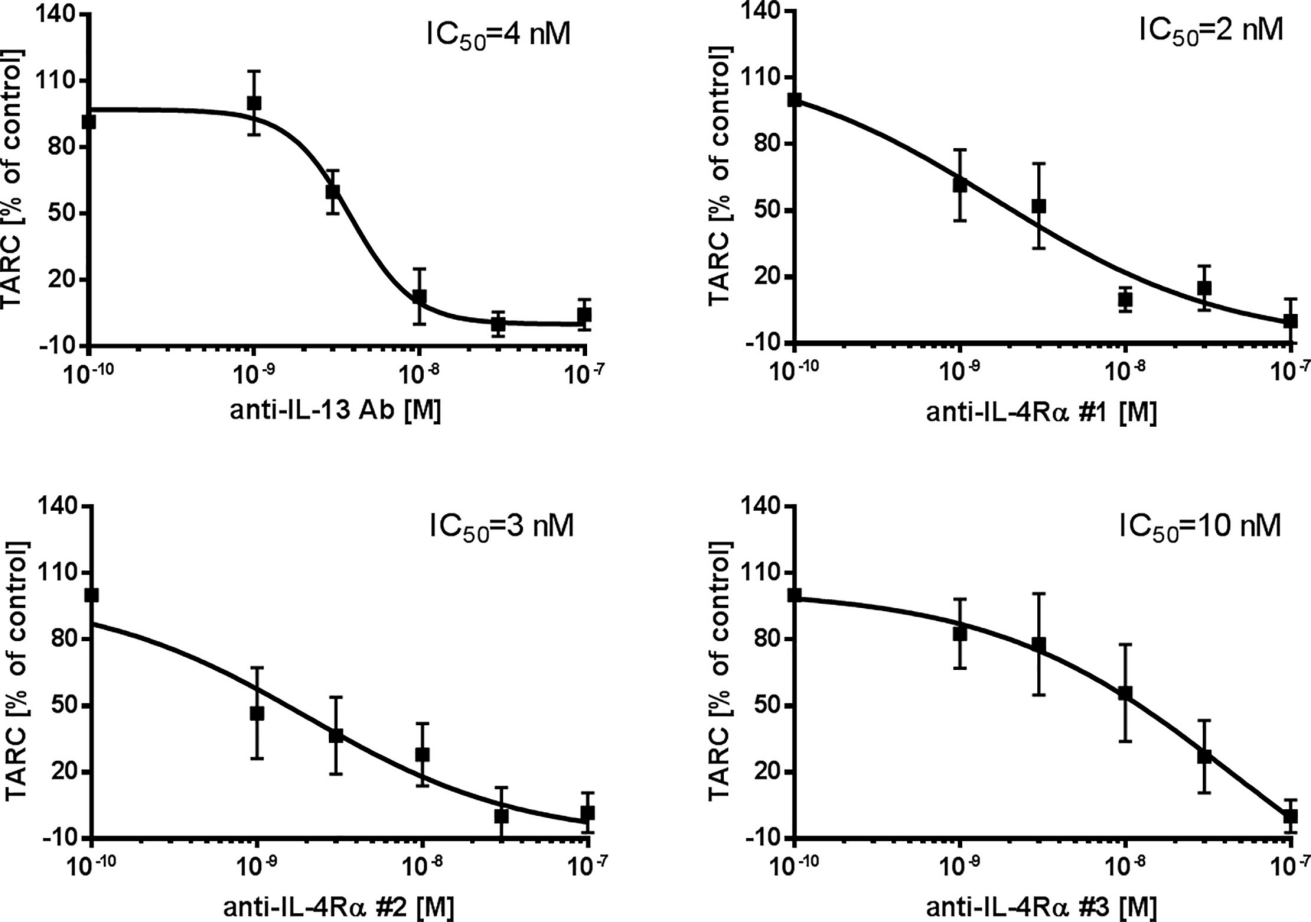
Inhibitory effects on IL-13-induced TARC production. Human PCLS were co-stimulated with 10 nM IL-13 and increasing concentrations of the antagonists a) anti-IL-13 Ab (n = 6), b) anti-IL-4Rα #1 (n = 5), c) anti-IL-4Rα #2 (n = 5), and d) anti-IL-4Rα #3 (n = 4). Results are displayed as the sum of intra and extracellular cytokines and normalized (as a percentage) to the untreated tissue control. Curve fitting was performed by non-linear regression analysis based on four parameters. Data are presented as mean ± SEM.

Treatment with anti-IL-13 Ab, anti-IL-4Rα Abs #1, #2 and Anticalin #3 resulted in dose-dependently reduced levels of IL-13-induced eotaxin-3 (Fig 5) and TARC (Fig 6). Tlc26 is a wildtype tear lipocalin, a negative control for anti-IL-4Rα #3, and revealed no effect on IL-13-induced eotaxin-3 and TARC release (S4 Fig). IC_50_ was used to describe the efficacy of the tested inhibitors. Inhibition of eotaxin-3 release was comparable for anti-IL-13 Ab (IC_50_ = 9 nM), anti-IL-4Rα #2 (IC_50_ = 6 nM), and anti-IL-4Rα #3 (IC_50_ = 5 nM). Anti-IL-4Rα #1 showed less attenuation potency (IC_50_ = 17 nM). Inhibition of TARC release was comparable for all four tested substances.

## Discussion

Asthma is defined by clinical features such as mucus hypersecretion, airway inflammation and airway hyperresponsiveness. IL-13 is considered to be a central molecule in asthma which can directly induce pathomechanisms leading to the disease development. However, most of the current knowledge on the biological mechanisms of IL-13 was generated in murine models. In our study we used a human-based *ex-vivo* organ tissue to assess the effect of IL-13 in comparison to commonly used laboratory animals and *in-vitro* cell cultures based on clinically relevant endpoints such as mucus hypersecretion, inflammation, and airway hyperresponsiveness.

Assessment of the direct effect of IL-13 on mucus hypersecretion in the *ex-vivo* human bronchial tissue confirmed mucin induction as shown by a two-fold increase compared with the control. These results are in line with previous *in-vivo* rodent studies. Perkins and colleagues, for example, showed that IL-13-deficient (IL-13^-/-^) mice failed to generate goblet cell hyperplasia, proving the critical role of the cytokine [32]. Moreover, intratracheal instillation of IL-13 in rats resulted in increased goblet cell density and mucin5AC protein expression [33]. But also *in-vitro*, IL-13 has already shown a direct effect on mucin5AC expression in primary bronchial epithelial cell culture [34]. Furthermore, Kondo *et al*. showed that elimination of IL-13 reverses established goblet cell metaplasia in primary epithelial cell culture [35]. All in all, we conclude that IL-13 directly stimulate mucus production in human bronchial tissue.

Airway inflammation is another hallmark in allergic asthma and is mediated by increased numbers of eosinophils and T lymphocytes. Cytokines such as eotaxin-3 and TARC impact activation, migration, and survival of these cells and can, therefore, be used as biomarkers of allergic airway inflammation. Both cytokines are released mainly by epithelial cells, endothelial cells, macrophages, T lymphocytes as well as dendritic cells. Any migration of cells from the blood and lymph fluid into the lung tissue and vice versa cannot be investigated in PCLS, while secretion of cytokines, which coordinates cell migration, can be examined in lung tissue *ex-vivo* [25, 26]. Human lung tissue exposed for 24 h to IL-13 showed pronounced release of eotaxin-3 and TARC. This observation is in agreement with Berkman *et al*., who reported elevated levels of eotaxin-3 24 h after allergen challenge in mild asthmatic patients and suggested a role of eotaxin-3 in the allergen-induced recruitment of eosinophils [36]. In addition, increased TARC levels were reported in serum and sputum of asthmatic patients [37]. Intratracheal administration of IL-13 to BALB/c mice resulted in an asthma phenotype *in-vivo* showing, for example, elevated levels of eosinophils and AHR 48 h after cytokine administration [38]. Elevated levels of eotaxin-3 and TARC have also been shown in previous studies using similar concentrations of IL-13 as in the present study (1 nM for primary bronchial epithelial cells [39] or endometrial epithelial cells [40] and 2 nM of IL-13 for A549 [41]).

It has been proposed that cytokine release and airway inflammation finally lead to AHR in asthmatics. AHR is characterized by an increased sensitivity and an excessive bronchoconstriction towards methacholine [42]. Although it is very useful in the clinics, showing association between AHR and increased risk of exacerbation, not all asthmatics react with AHR following allergen challenge [43]. The pathomechanism of AHR remains unknown and the implication of airway inflammation and structural airway remodeling require further investigation. In this context, the direct contribution of IL-13 on AHR was evaluated in PCLS, as previously asthmatic features such as mucus secretion and inflammation were well reflected *ex-vivo*. Induction of AHR by IL-13 has frequently been shown in rodent models and has also been confirmed in studies using human PCLS. The study by Cooper *et al*. showed increased airway sensitivity of human PCLS to carbachol after IL-13 treatment for 18 h [31]. In our study, however, IL-13 did not induce AHR in human PCLS. Moreover, we have not been able to observe AHR in mouse, rat or marmoset PCLS pre-incubated for 18 h with IL-13. The general approach of Cooper and colleagues was comparable to ours. They used PCLS of healthy human donors containing perpendicular airways of ≤ 1 mm diameter, incubated them with 100 ng/mL (8 nM) IL-13 for 18 h and treated these with carbachol at increasing concentrations from 10^−8^ to 10^−4^ M to induce airway contraction. The differences in results could be due to different airway responsiveness depending on airway size. Airway size in particular could be a reason for the differences in the results from those obtained by Cooper *et al*., 2009. Human airways with a diameter less than 2 mm correspond to generation 11 in the lung and are defined as terminal small airways [44, 45]. The data obtained in our study were distinguished according to airway size. Human PCLS with an airway diameter of 500 μm to 1300 μm were separated from PCLS with an airway size below 400 μm. As previously shown, contraction depends on airway size [45]. Airways with a diameter smaller than 400 μm close completely, in contrast to larger airways. Therefore, similar airway sizes were selected for control and treatment to avoid misleading interpretation of data. We did not observe responder vs. non-responder, unless the size distribution in the data set was not comparable.

The hypothesis was that IL-13 either directly or indirectly changes the contractile signaling of airway smooth muscle cells. It is known that IL-13 signals through the STAT6 pathway. The time frame for phosphorylation was shown to be only 15 min [46]. Other research groups have shown that IL-13 can directly lead to calcium fluxes with no increase of muscarinic receptor density [47]. In mouse *in-vivo* studies AHR was observed already after 6 h [48]. Based on that, we assumed that IL-13-induced hyperresponsiveness occurs shortly after exposure. In previous studies we observed that methacholine-induced airway constriction measured in PCLS decreases over time [49]. To avoid misinterpretation of the data, shorter time points for IL-13 stimulation on the day after the slicing procedure were chosen (0.5 h, 3 h and 6 h). This revealed AHR exclusively in rodent but not in marmoset or human PCLS. Murine PCLS exhibited IL-13-induced AHR after six hours, which is comparable to the *in-vivo* situation [48]. We observed that IL-13 changes the responsiveness of murine airways to methacholine already after 30 min, before the pro-inflammatory tissue response with recruiting cytokine and chemokine release reaches its maximum. This leads to the assumption that IL-13 possesses a direct effect on airway smooth muscles (ASM) without inflammation as the inducing key event. In general, the presence of IL-13 receptor on ASM of lung tissue has been well known for many years. ASM cells of *ex-vivo* lung tissue are also positive for the IL-13Rα1 receptor, which is usually expressed with an IL-4Rα chain [12]. In addition, previous studies showed receptor presence on cardiac and vascular smooth muscles [50] and IL-13 was hypothesized to directly enhance Ca^2+^ influx in ASM. Nevertheless, the exact mechanisms leading to AHR as well as the involvement of IL-13 in this system have not been completely understood so far. Since exclusively rodents showed AHR, this observation for the first time confirms species-specific differences in the biological mechanism of IL-13 between laboratory animals and humans. Our data showed no direct effect of IL-13 on airway smooth muscle cells of healthy human tissue. This result confirms the findings from clinical studies showing that blocking of IL-13 by e.g. Lebrikizumab is not sufficient to significantly improve FEV_1_ [51]. This emphasizes the complexity of AHR and shows that inhibition of single molecules is insufficient to improve lung function. This pronounces the impact that airway remodeling has on airway hyperresponsiveness.

On the other hand, attenuation of locally produced cytokines is currently a focus of research on targeted therapies [52, 53] and can be a new approach in personalized medicine. Therefore, inhibitors directed against IL-13 or IL-4Rα have been developed as new asthma therapeutics. In the present study, we have shown that profiling or screening of inhibitors can also be performed in *ex-vivo*-based assays using biomarkers such as eotaxin-3 and TARC. We were able to show that the anti-IL-13 Ab (similar to Lebrikizumab) attenuated eotaxin-3 and TARC release. This is in line with clinical evidence, where Lebrikizumab showed positive therapeutic effects on baseline spirometry in patients with chronic moderate to severe asthma [16]. Another antibody targeting the soluble IL-13 to prevent receptor binding to both IL-13Rα1 and IL-13Rα2 by, for example, GSK679586 (GlaxoSmithKline, Middlesex, UK) showed no significant improvement in asthma control in patients with severe asthma in a clinical trial [54]. Moreover, specific binding of tralokinumab (earlier CAT-354, AstraZeneca, Södertälje, Sweden), another anti-IL-13 antibody, was approved with regard to its safety and tolerability profile, but it showed treatment effects only in a defined group of patients [55]; a phase-III clinical trial has been completed (NCT02161757). On the other hand, several other studies indicated that inhibition of the IL-4 and IL-13 pathways represents a more promising approach. Therefore, an anti-IL-4Rα #1 (similar to AMG 317) was tested in PCLS and was less effective in inhibiting IL-13 stimulation of human lung tissue compared with anti-IL-13 Ab (similar to Lebrikizumab). The subcutaneous doses administered in a clinical study were up to 300 mg per patient [18], which peaked after 49 h with 9.59 μg/mL in the plasma [56]. This corresponds to a concentration of 60 nM. Our data, however, indicate that even at this concentration, AMG 317 is ineffective in attenuating eotaxin-3 and TARC secretion. This could be one of the reasons for its reduced efficacy, as only a subset of patients in the study showed a treatment effect. Anti-IL-4Rα #2 (similar to REGN668/Dupilumab) showed comparable efficacy in reducing eotaxin-3 and TARC secretion as anti-IL-13 Ab (similar to Lebrikizumab). Dupilumab has been tested in a phase-II study in patients with persistent asthma and elevated eosinophil levels. Eotaxin-3 and TARC levels decreased one week after the first administration and remained lower than baseline over the 12 weeks of the study. These results confirmed the biological efficacy of the antagonist, however, it was inefficient in reducing peripheral blood eosinophils [57, 58]. The new antagonist anti-IL-4Rα #3 (Anticalin PRS-060) also significantly reduced eotaxin-3 and TARC secretion and showed comparable efficacy to other inhibitors in our study. A closer look at the shape of the curve for anti-IL-13 Ab shows that it has a different shape compared to the others. The assumed mechanism is that it directly binds to IL-13, removing the cytokine by neutralization. The curves for anti-IL-4 Rα show different dose-response slopes. The proposed mechanism is that the anti-IL-4Rα Abs block the recruitment of IL-4Rα to the binary IL-13/IL-13Rα1 complex on cell surface.

To conclude, this study showed that IL-13 induces the secretion of biomarkers of allergic inflammation, such as TARC and eotaxin-3, as well as mucus hypersecretion in human lung tissue *ex-vivo*. The presence of IL-13 only and the subsequent release of pro-inflammatory cytokines were–without airway remodeling–not sufficient to induce significant changes in the sensitivity of human airway smooth muscle cells as IL-13 induced no AHR in healthy human lung tissue *ex-vivo*. Concerning this issue, the mechanisms in laboratory rodents seems to be different, as IL-13 efficiently induced AHR in commonly used rodents *ex-vivo*. This observation highlights differences in mechanisms of IL-13 in rodents when compared to human. By adding results from highly relevant human tissue, mechanistic understanding of the activity of IL-13 was extended and its efficacy in humans was demonstrated.

## Supporting information

S1 Fig **a) Mucus overproduction induced by 10 nM IL-13 for 20 h in human bronchus tissue. Results for mucin5AC secretion were normalized for weight of bronchus tissue.** Data are presented as mean±SEM, n = 3, *p<0.05 (Wilcoxon signed-rank test). b) Representative fluorescence immunostaining of mucin5AC in human bronchus. Red color shows epithelial cells by pan-cytokeratin labeled with Alexa 647 and yellow color shows mucin5AC labeled with Cy3. Mucin5AC is located behind the epithelium in exocrine glands. c) Distribution of mucus producing goblet cells in large (>2mm) and small (<2mm) airways. Embedded human airways were quantified for PAS-positive cells and normalized to the epithelial perimeter. No statistically difference was observed for the frequency of mucus-producing cells in big and small airways. For big airways (>2mm) is n = 3 and for small airways (<2mm) is n = 5.
(TIF)

S2 Fig Absolute values of IL-13 induced eotaxin-3 and TARC secretion in human PCLS. Lung tissue was stimulated with rhIL-13 for 24 h. Eotaxin-3 and TARC were determined in supernatant and tissue lysate. Data are presented as mean±SEM, a) n = 7 and b) n = 8, *p<0.05, **p<0.01; ***p<0.001 compared to untreated tissue control, Friedman test and Dunn’s Multiple Comparison Post-hoc test.
(TIF)

S3 Fig **Effect of IL-13 on methacholine-induced airway constriction in a) murine, b) rat, c) marmoset, and d) human PCLS after 18 h.** PCLS were pre-incubated with 8 nM IL-13 for 18 h before stimulation with increasing concentrations of methacholine. Airway constriction was determined as a percentage of the initial airway area. Data are presented as mean ± SEM; n = 4.
(TIF)

S4 Fig Wild type lipocalin (TLC26) has no inhibitory effect on IL-13 induced eotaxin-3 and TARC production in human PCLS. Lung tissue was co-stimulated with 10 nM rhIL-13 and increasing concentration of TLC26 for 24 h. Secretion of a) eotaxin-3 and b) TARC were measured in supernatant and tissue lysate. Data were normalized to the tissue control (in percent) and are presented as mean±SEM, n = 4.
(TIF)
