# Supplementary figures and images for: Human lung tissue provides highly relevant data about efficacy of new anti-asthmatic drugs
Source: PLoS One. 2018 Nov 30;13(11):e0207767. doi: 10.1371/journal.pone.0207767 (PMC6267969; doi:10.1371/journal.pone.0207767)

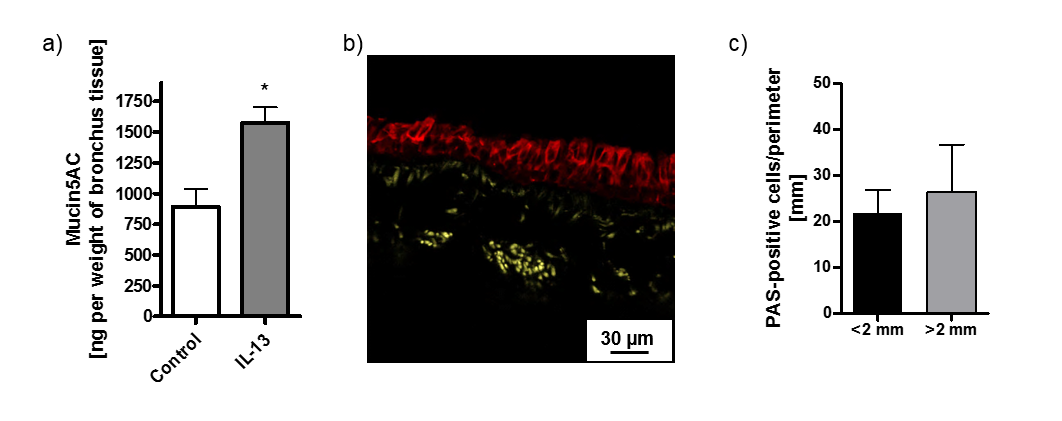

Supplement: S1 Fig — a) Mucus overproduction induced by 10 nM IL-13 for 20 h in human bronchus tissue. Results for mucin5AC secretion were normalized for weight of bronchus tissue. Data are presented as mean±SEM, n = 3, *p<0.05 (Wilcoxon signed-rank test). b) Representative fluorescence immunostaining of mucin5AC in human bronchus. Red color shows epithelial cells by pan-cytokeratin labeled with Alexa 647 and yellow color shows mucin5AC labeled with Cy3. Mucin5AC is located behind the epithelium in exocrine glands. c) Distribution of mucus producing goblet cells in large (>2mm) and small (<2mm) airways. Embedded human airways were quantified for PAS-positive cells and normalized to the epithelial perimeter. No statistically difference was observed for the frequency of mucus-producing cells in big and small airways. For big airways (>2mm) is n = 3 and for small airways (<2mm) is n = 5. (TIF) [file pone.0207767.s001.tif]

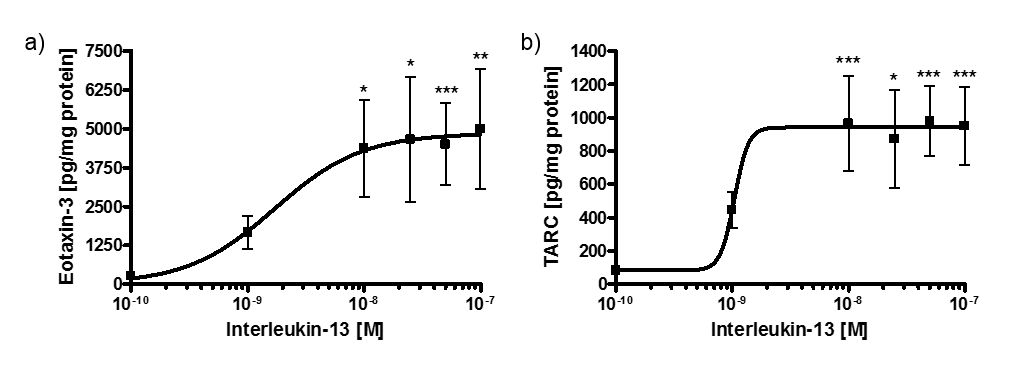

Supplement: S2 Fig — Lung tissue was stimulated with rhIL-13 for 24 h. Eotaxin-3 and TARC were determined in supernatant and tissue lysate. Data are presented as mean±SEM, a) n = 7 and b) n = 8, *p<0.05, **p<0.01; ***p<0.001 compared to untreated tissue control, Friedman test and Dunn’s Multiple Comparison Post-hoc test. (TIF) [file pone.0207767.s002.tif]

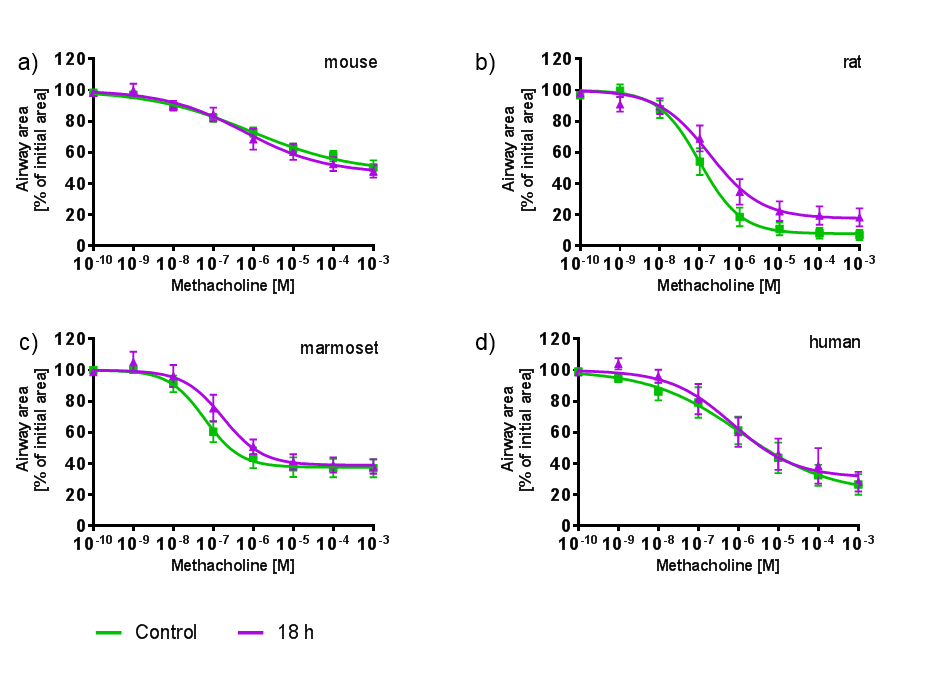

Supplement: S3 Fig — Effect of IL-13 on methacholine-induced airway constriction in a) murine, b) rat, c) marmoset, and d) human PCLS after 18 h. PCLS were pre-incubated with 8 nM IL-13 for 18 h before stimulation with increasing concentrations of methacholine. Airway constriction was determined as a percentage of the initial airway area. Data are presented as mean ± SEM; n = 4. (TIF) [file pone.0207767.s003.tif]

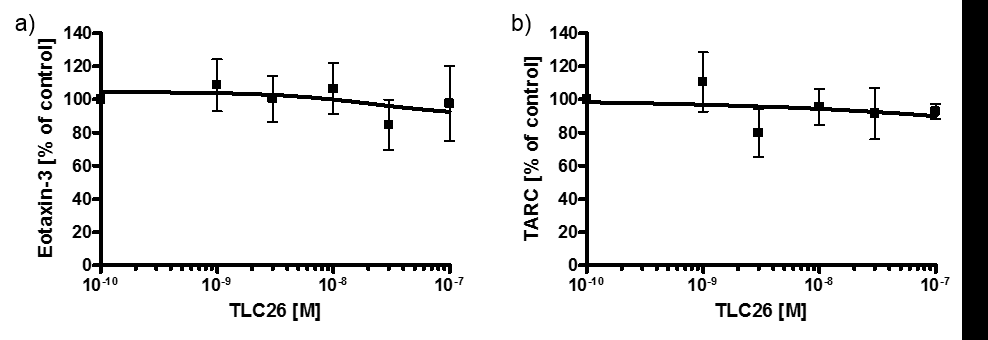

Supplement: S4 Fig — Lung tissue was co-stimulated with 10 nM rhIL-13 and increasing concentration of TLC26 for 24 h. Secretion of a) eotaxin-3 and b) TARC were measured in supernatant and tissue lysate. Data were normalized to the tissue control (in percent) and are presented as mean±SEM, n = 4. (TIF) [file pone.0207767.s004.tif]
